# Prediction of late left ventricular thrombus formation after ST-elevation myocardial infarction: A cardiovascular magnetic resonance study

**DOI:** 10.1016/j.ijcha.2025.101753

**Published:** 2025-07-19

**Authors:** Magdalena Holzknecht, Christina Tiller, Ivan Lechner, Fritz Oberhollenzer, Alex Kaser, Martin Reindl, Felix Troger, Mathias Pamminger, Axel Bauer, Bernhard Metzler, Sebastian Johannes Reinstadler, Agnes Mayr

**Affiliations:** a University Clinic of Internal Medicine III, Cardiology and Angiology, Medical University of Innsbruck, Anichstrasse 35, A-6020 Innsbruck, Austria; b University Clinic of Radiology, Medical University of Innsbruck, Anichstrasse 35, A-6020 Innsbruck, Austria

**Keywords:** Left ventricular thrombus, ST-elevation myocardial infarction, Risk score, Magnetic resonance imaging

## Abstract

**Background:**

Little is known about the prevalence of late left ventricular thrombus (LVT) formation after ST-elevation myocardial infarction (STEMI). Compared with transthoracic echocardiography (TTE), cardiovascular magnetic resonance (CMR) imaging has a much higher sensitivity for LVT detection in patients after acute STEMI. However, routine CMR imaging is currently not integrated in post-STEMI management. We sought to develop a practical risk score for the prediction of late LVT formation after STEMI.

**Methods and Results:**

Six hundred and nine patients with STEMI underwent CMR at 3 [IQR:2–4] days and 4.4 [IQR:4.1–4.9] months after primary percutaneous coronary intervention (PCI) for acute STEMI. A LVT was visualized in 37 patients (6.1 %) at baseline CMR and was newly diagnosed in 10 patients (1.6 %) at 4 months follow-up (4FU) CMR and in 4 patients (0.7 %, 60 % false-negative rate of TTE in detecting LVT) by 4FU TTE. A simple clinical risk score including associates of late LVT (left anterior descending artery as culprit vessel and 4FU NT-pro-BNP > 236 ng/l), with a range of 0 to 2 points (median risk score: 1 point) showed a strong and significantly higher area under the curve (0.89, 95 %CI 0.86–0.91; p < 0.001) for LVT prediction at 4FU than each individual risk factor alone (p < 0.001). The sensitivity and specificity of the risk score were 100 % and 77 %, respectively.

**Conclusions:**

The proposed risk score may offer preliminary utility in predicting late LVT and could help to identify patients with STEMI in whom CMR for late LVT assessment might be particularly informative. Additional investigation in larger cohorts is warranted to validate the clinical application of the score.

## 1 Introduction

In the early phase after ST-elevation myocardial infarction (STEMI) treated with primary percutaneous coronary intervention (PCI), left ventricular thrombus (LVT) formation is still a frequent complication with an incidence of 4–6 % [1,2]. However, little is known about LVT occurring at later time points, as serial imaging with high sensitivity for LVT detection is rare [3,4].

Cardiovascular magnetic resonance (CMR) imaging represents the optimal imaging modality for the detection (sensitivity: 87 %, specificity: 99 %) and characterization of LVT [[5], [6], [7], [8]] and should be considered in patients with high clinical suspicion of LVT or in patients with equivocal echocardiographic images [9]. Several authors described a limited sensitivity of transthoracic echocardiography (TTE) for LVT detection with a sensitivity of 32–35 % for non-contrast TTE and a sensitivity of 64 % for contrast-enhanced TTE [1,[6], [7], [8],^10^]. However, CMR imaging is currently not integrated in routine care after STEMI and therefore a relevant number of LVT are likely to remain undetected [11,12]. Identifying simple and effective strategies enabling reliable detection rates of LVT post-STEMI remains therefore desirable.

We aimed to develop an easily applicable risk score by evaluating culprit lesion location and biomarker profiles at 4 months follow-up (4FU) for the prediction of late LVT after STEMI. Hereby, patients at elevated risk could be identified and referred to CMR evaluation to improve late LVT detection and management.

## 2 Methods

### 2.1 Patient population and endpoint definitions

Patients enrolled in the prospective observational “Magnetic Resonance Imaging In Acute ST-Elevation Myocardial Infarction (MARINA-STEMI)” study [[13], [14], [15]] (NCT04113356) between 2011 and 2022 were evaluated for inclusion in the final analysis. The following inclusion criteria were applied: first STEMI according to the European Society of Cardiology/American College of Cardiology committee criteria [16,17], revascularization by primary PCI within 24 h after onset of ischemic signs or symptoms and Killip class < 3 at the time of CMR imaging. Exclusion criteria were defined as inability or unwillingness to sign written informed consent, an age < 18 years, any history of a previous myocardial infarction or coronary intervention, an estimated glomerular filtration rate < 30 mL/min per 1.73 m^2^ and any other contraindication to CMR examination (pacemaker, severe claustrophobia, orbital foreign body, cerebral aneurysm clip, or known or suggested contrast agent allergy to gadolinium).

The study was designed and conducted in compliance with the Declaration of Helsinki and received approval by the research ethics committee of the Medical University of Innsbruck.

The primary endpoint was the presence of LVT, detected by CMR, 4 months after acute STEMI treated with primary PCI.

### 2.2 Biochemical measurements

Biochemical measurements of high-sensitivity (hs) cardiac troponin T (cTnT), C-reactive protein (CRP) and N-terminal prohormone of brain natriuretic peptide (NT-pro-BNP) were performed serially at baseline (BL) as well as at 4FU. Measurements of hs-CRP were conducted on the cobas® 8000 modular analyzer (Roche Diagnostics®, Vienna, Austria) and concentrations of hs-cTnT were determined applying a validated enzyme immunoassay (hs-cTnT; E170, Roche Diagnostics®, Vienna, Austria). NT-pro-BNP levels were measured using a commercially available electrochemiluminescence immunoassay (ECLIA Roche Diagnostics®, Vienna, Austria).

### 2.3 Transthoracic echocardiography

TTE was performed at BL within the first week after PCI at the discretion of the treating physician. To analyze the diagnostic accuracy of TTE in thrombus detection, patients with new-onset LVT on 4FU CMR were referred to a hospital echocardiographer blinded to the CMR result to screen for LVT. Standard post-processing software was applied (Image Arena, TOMTEC Imaging Systems, Unterschleissheim, Germany). The diagnostic algorithm for LVT detection was published in a previous work [8]. The use of contrast agent was at the discretion of the examiner. Echocardiographic analyses were conducted by two experienced cardiologists with an EACVI Certification in Adult Transthoracic Echocardiography (TTE), blinded to clinical and CMR data.

### 2.4 Cardiovascular magnetic resonance imaging

All patients were investigated in supine position on a 1.5 Tesla clinical MR scanner (MAGNETOM Avantofit; Siemens Healthineers AG, Erlangen, Germany) within the first week after treatment with PCI (BL) and at 4FU. The standardized imaging protocol of our research group was published in detail in advance [15,18].

High-resolution cine images (CINE voxel resolution: 1.5 mm x 1.5 mm x 6.0 mm) in long- and short axis covering the LV [[10], [11], [12]] were obtained using a balanced steady state free precession (bSSFP) sequence with retrospective electrocardiographic (ECG) gating. For the determination of LV volumes and LV ejection fraction (LVEF), standard postprocessing software (Circle Cardiovascular Imaging, Calgary, Canada) was applied with semi-automatic detection of LV endo- and epicardial borders. ECG-triggered, phase-sensitive inversion recovery sequences were used to obtain late gadolinium enhancement (LGE) images (LGE voxel resolution: 1.4 mm x 1.4 mm x 8.0 mm) 15–20 min after application of a 0.2 mmol/kg bolus of contrast agent (Gadovist®, Bayer, Leverkusen, Germany). For quantification of infarct size (IS), a PACS workstation (IMPAX®, Agfa HealthCare, Bonn, Germany) was used, whereas “hyperenhancement” was defined as + 5 standard deviations above the signal intensity of remote LV myocardium [19,20]. IS was expressed as percentage of total LV myocardial mass (LVMM). Microvascular obstruction (MVO) was defined as persisting area of “hypoenhancement” within the hyperenhanced territory and was also reported as percentage of LVMM [21]. Regions of MVO were included in aggregate IS.

The diagnostic pathway for the detection of LVT was published in a previous work [22]. In brief, LVT was defined as an intracavitary mass with low-intensity tissue characteristics on LGE images, delineated by high-signal intensity structures (e.g. infarcted myocardium and blood pool) irrespective of location or morphology. LVT was carefully distinguished from MVO, papillary muscles, trabeculae, chordal structures, tangential views of the LV wall and technical artefacts [2]. If accurate differentiation between MVO and wall-adherent thrombus was uncertain, LGE sequence was repeated using a “long-inversion” time of ∼ 550 ms. If the diagnosis still remained unclear, the LGE sequence was repeated serially to detect MVO by stepwise filling it with contrast medium. This was continued until the differentiation between these entities was definitely clarified [8]. The presence or absence of LVT on CMR was evaluated by 2 experienced observers (Euro CMR Level 2), blinded to each other’s results and to the clinical data. In case of disagreement, consensus was achieved. LVT location, volume and morphology (flat wall-adherent, mass-like/intracavitary and mixed) were evaluated [23]. Late LVT was defined as the occurrence of new LVT at 4FU CMR [4].

LV aneurysm was determined at 4FU CMR and was defined as a dyskinetic protrusion of the LV contour in diastole and systole [10].

Incomplete protocol was defined as premature termination of the investigation due to patient, medical or technical concerns.

### 2.5 Statistical analyses

Continuous data are expressed as median with interquartile ranges (IQR) and categorical variables are presented as numbers with corresponding percentages. Differences in continuous and categorical variables between two groups were tested by Mann–Whitney *U* test and Chi-square test, respectively.

Receiver operating characteristic (ROC) analyses were applied for biomarkers at 4FU and culprit lesion location, which were significantly associated with new 4FU LVT in Table 1, to evaluate the area under the curve (AUC) for the prediction of new 4FU LVT. Youden Index was calculated to evaluate the optimal cut-off value for dichotomization of the continuous LVT predictor [24]. The dichotomized biomarker predictor of new LVT at 4FU and the variable left anterior descending artery (LAD) as culprit vessel were included in the risk score. Each variable received one score point. The population was classified into 2 risk categories: at low risk (0 to1 point) and at elevated risk (2 points). ROC-curves were compared according to DeLong et al. [25]. Following Rice and Harris, AUC values were categorized as negligible (≤0.55), small (0.56–0.63), moderate (0.64–0.70) and strong (≥0.71) [26]. To evaluate the incremental prognostic benefit of NT-pro-BNP above the cut-off in addition to the culprit lesion, net reclassification improvement (NRI) was calculated by using the R package “PredictABEL”. All tests were 2-tailed and the significance level was set at 0.05. SPSS Statistics 29.0 (IBM, Armonk, NY, USA), MedCalc Statistical Software version 19.4.1 (MedCalc Software Ltd, Ostend, Belgium) and R Statistical Software version 4.1.2 (R Core Team 2021) were used for statistical analyses. Fig. 2 was created with BioRender.com.

**Table 1:** Patient characteristics according to the presence and absence of baseline left ventricular thrombus and new left ventricular thrombus at 4 months follow-up.

**Characteristic**	**BL LVT**				**4FU LVT**	
	**Total population (n = 609)**	**Absence** **(n = 572, 93.9 %)**	**Presence** **(n = 37, 6.1 %)**	**p-value**	**New** **(n = 10, 1.6 %)**	**p-value**
**BL age, years**	57[51–66]	58[51–66]	55[51–64]	0.497	51[45–69]	0.374
**Assigned female at birth, n (%)**	113(19)	106(19)	7(19)	0.953	3(30)	0.348
**BL body mass index, kg/m^2^**	26.1[24.3–28.4]	26.1[24.2–28.4]	27.1[24.4–31.1]	0.210	24.6[23.2–26.1]	0.095
**BL hypertension, n (%)**	272(45)	258(45)	14(38)	0.389	5(50)	0.732
**BL active cigarette smoking, n (%)**	341(56)	322(56)	19(51)	0.557	5(50)	0.700
**BL hyperlipidemia, n (%)**	320(53)	304(53)	16(43)	0.242	5(50)	0.871
**BL diabetes mellitus, n (%)**	43(7)	38(7)	5(14)	0.114	0(0)	0.379
**BL family history, n (%)**	187(31)	176(31)	11(30)	0.894	2(20)	0.459
**Time from symptom onset to PCI, min**	185[112–329]	182[112–324]	240[124–436]	0.109	224[124–255]	0.931

**Culprit lesion, n (%)**				**<0.001**		**0.021**
**LM**	3(0.5)	3(0.5)	0(0)		0(0)	
**RCA**	235(38.5)	233(40.8)	2(5.4)		0(0)	
**LAD**	285(46.8)	250(43.7)	35(94.6)		10(100)	
**LCX**	82(13.5)	82(14.3)	0(0)		0(0)	
**RI**	4(0.7)	4(0.7)	0(0)		0(0)	

**Number of affected vessels, n (%)**				**0.013**		0.451
**1**	353(58)	323(56)	30(81)		7(70)	
**2**	176(29)	171(30)	5(14)		3(30)	
**3**	80(13)	78(14)	2(5)		0(0)	

**TIMI flow pre-PCI, n (%)**				0.217		0.133
**0**	393(64.6)	366(64.0)	27(73.0)		10(100)	
**1**	90(14.8)	83(14.5)	7(18.9)		0(0)	
**2**	88(14.4)	85(14.9)	3(8.1)		0(0)	
**3**	38(6.2)	38(6.6)	0(0)		0(0)	

**TIMI flow post-PCI, n (%)**				**<0.001**		**<0.001**
**0**	3(0.5)	2(0.35)	1(2.7)		0 (0)	
**1**	5(0.8)	2(0.35)	3(8.1)		0 (0)	
**2**	55(9.0)	51(8.9)	4(10.8)		5 (50)	
**3**	546(89.7)	517(90.4)	29(78.4)		5 (50)	

**BL peak hs-cTnT, ng/l**	4857[2145–8012]	4673[2082–7549]	7592[4931–13903]	**<0.001**	11491[7013–17814]	**<0.001**
**BL peak hs-CRP, mg/l**	25[13–49]	23[12–45]	82[33–14]	**<0.001**	30[13–54]	0.739
**BL NT-pro-BNP, ng/l**	1330[691–2442]	1257[674–2254]	3285[2076–5825]	**<0.001**	1691[962–3621]	0.215
**4FU hs-cTnT, ng/l**	10[7–15]	10[7–14]	13[10–19]	**<0.001**	12 [7–19]	0.605
**4FU hs-CRP, mg/l**	1.0[0.6–2.1]	1.0[0.6–2.1]	1.1[0.6–2.6]	0.652	1.2[0.6–2.5]	0.712
**4FU NT-pro-BNP, ng/l**	210[107–450]	204[101–416]	516[217–1000]	**<0.001**	530[374–829]	**0.002**
**Atrial fibrillation, n (%)**	17(3)	16(3)	1(3)	0.700	0(0)	0.738

**Discharge medication (index event)**						
**Oral anticoagulation, n (%)**	49(8)	17(3)	32(87)	**<0.001**	0(0)	0.346
**Aspirin, n (%)**	603(99)	568(99)	35(95)	**0.005**	10(100)	0.750
**Dual antiplatelet therapy, n (%)**	603(99)	567(99)	35(95)	0.012	10(100)	0.731
**Βeta-blockers, n (%)**	557(92)	521(91)	36(97)	0.190	10(100)	0.330
**ACE-inhibitors/AT1-antagonists, n (%)**	577(95)	542(95)	35(95)	0.220	10(100)	0.324
**Statins, n (%)**	605(99)	568(99)	37(100)	0.610	10(100)	0.795

**4FU medication**						
**Oral anticoagulation, n (%)**^a^	40(7)	26(5)	14(38)	**<0.001**	0(100)	0.398
**Aspirin, n (%)**	581(95)	558(98)	23(62)	**<0.001**	10(100)	0.484
**Dual antiplatelet therapy, n (%)**	538(88)	515(90)	23(62)	**<0.001**	10(100)	0.511
**Βeta-blockers, n (%)**	522(86)	485(85)	37(100)	**0.010**	8(80)	0.603
**ACE-inhibitors/AT1-antagonists, n (%)**	529(87)	499(87)	30(81)	0.639	9(90)	0.672
**Statins, n (%)**	584(96)	548(96)	36(97)	0.657	10(100)	0.509

BL = baseline, 4FU = 4 months follow-up, BL = baseline, Hs-CRP = high sensitivity C-reactive protein, hs-cTnT = high sensitivity cardiac troponin T, LAD = left anterior descending artery, LCX = left circumflex artery, LVT = left ventricular thrombus, NT-pro-BNP= N-terminal prohormone of brain natriuretic peptide, PCI = percutaneous coronary intervention, RCA = right coronary artery, RI = ramus intermedius, TIMI = thrombolysis in myocardial infarction.

a prior to 4FU CMR.

**Fig. 1:**
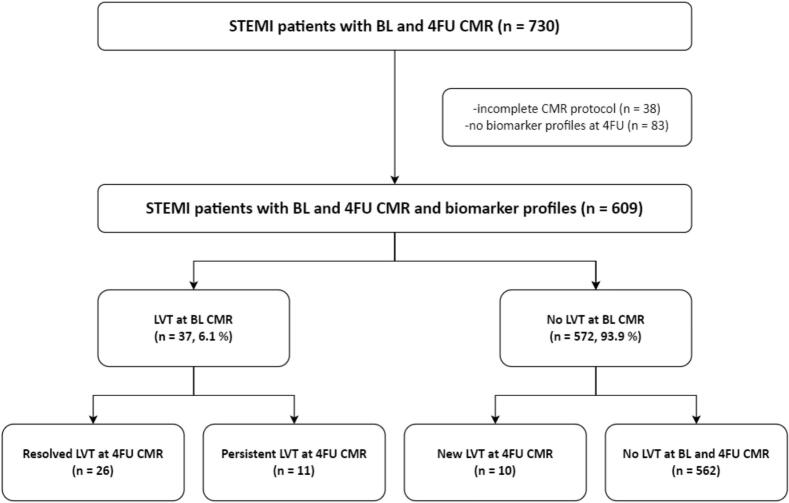
Flow chart of the study cohort. 4FU = 4 months follow-up, BL = baseline, CMR=Cardiovascular magnetic resonance, LVT = left ventricular thrombus, STEMI=ST-elevation myocardial infarction.

**Fig. 2:**
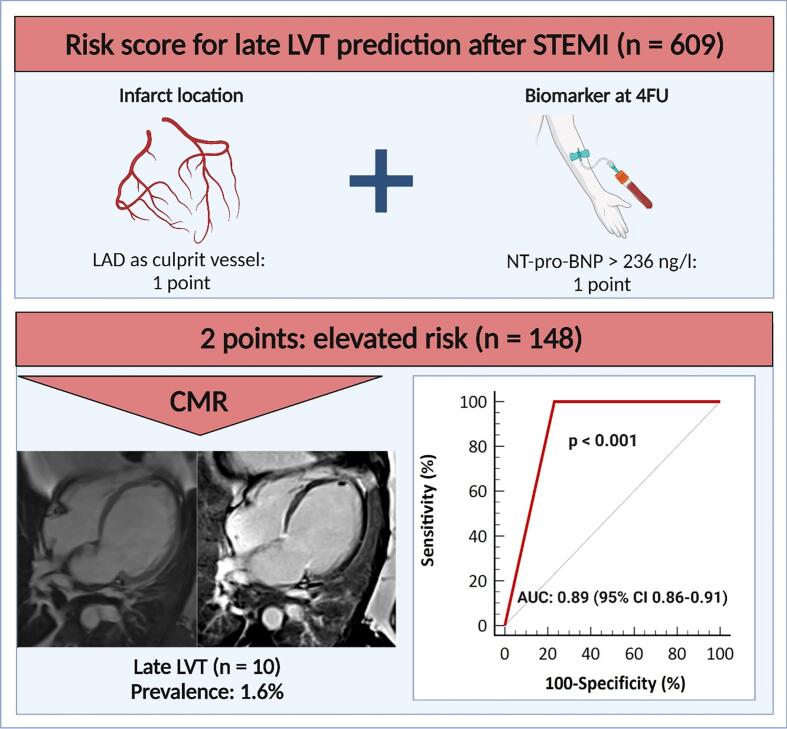
Risk score for the prediction of late LVT formation after STEMI. Based on the risk score, the population was categorized into 2 risk categories: at low (0-1point) and at elevated risk (2 points). The AUC of the risk score was 0.89 (95 % CI 0.86–0.91; p < 0.001) for the prediction of LVT. AUC = area under the curve, CI = confidence interval, LAD = left anterior descending artery. Other abbreviations as in Fig. 1. The figure was created with BioRender.com.

## 3 Results

### 3.1 Patient characteristics

Six hundred and nine patients were included in final analysis. Fig. 1 provides a study flow chart. Table 1 presents baseline characteristics of the overall cohort (n = 609) and separately for patients with (n = 37, 6.1 %) and without BL LVT (n = 572, 93.9 %), as well as for patients with new LVT at 4FU (n = 10, 1.6 %).

The culprit vessel was the LAD in all patients with new LVT at 4FU, whereas 2 patients with BL LVT (5.4 %) showed the culprit lesion in the right coronary artery. A significantly lower post-PCI thrombolysis in myocardial infarction (TIMI) flow was found in the LVT groups (all p < 0.001). Patients with BL LVT had more often single vessel disease (p = 0.013). Moreover, concentrations of peak hs-cTnT, peak hs-CRP and peak NT-pro-BNP were significantly related to BL LVT (p < 0.001). Regarding biomarkers collected at 4FU, only NT-pro-BNP was associated with late LVT (p = 0.002).

Anticoagulation was initiated at the discretion of the treating physician. Thirty-two patients (87 %) received anticoagulation after BL LVT detection by CMR. The reason for not starting anticoagulation directly in 5 of 37 patients (13 %) with BL LVT was the delayed receipt of CMR reports. The patients or the attending physician were informed immediately after communication of the CMR findings in order to ensure appropriate treatment.

All patients with late LVT received TTE at 4FU, whereas the false negative rate of TTE was 60 % in detecting LVT.

### 3.2 Cardiovascular magnetic resonance imaging findings

The main findings from CMR investigations are listed in Table 2. CMR was performed at a median of 3 [IQR: 2–4] days and at a median of 4.4 [IQR: 4.1–4.9] months after PCI for STEMI. Time to CMR did not differ between the overall cohort and patients with BL LVT (p = 0.197) and patients with new LVT at 4FU (p = 0.183). BL CMR diagnosed LVT in 37 patients (6.1 %). BL LVT resolved in 26 patients (70 %) and persisted in 11 patients (30 %) as assessed by 4FU CMR. New LVT was detected in 10 patients (1.6 %) by 4FU CMR and in 4 patients (0.7 %) by 4FU TTE, this means that 60 % of the CMR LVT were TTE-occult.

**Table 2:** CMR parameters according to the presence and absence of baseline left ventricular thrombus and new left ventricular thrombus at 4 months follow-up.

**Characteristic**	**BL LVT**				**4FU LVT**	
	**Total population (n = 609)**	**Absence** **(n = 572, 93.9 %)**	**Presence** **(n = 37, 6.1 %)**	**p-value**	**New** **(n = 10, 1.6 %)**	**p-value**
**Time to BL CMR, days**	3[2–5]	3[2–5]	4[3–6]	0.197	3[2–4]	0.183
**Time to 4FU CMR, days**	134[123–151]	134[124–151]	131[120–155]	0.421	129[117–145]	0.283
**BL LVEDV, ml**	167[139–191]	165[139–190]	189[161–212]	**<0.001**	169[138–190]	0.983
**BL LVESV, ml**	85[65–104]	83[65–101]	116[91–131]	**<0.001**	84[75–111]	0.600
**BL LVEF, %**	49[42–55]	49[43–55]	40[32–44]	**<0.001**	44[39–53]	0.157
**BL IS, % of LVMM**	15.9[8.2–25.5]	15.6[7.7–24.8]	25.2[16.5–31.9]	**<0.001**	28.5[22.3–36.2]	**<0.001**
**BL MVO, n (%)**	360(59)	330(58)	30(81)	**0.005**	10(100)	**0.008**
**BL MVO, % of LVMM**	0.4[0.0–2.2]	0.4[0.0–2.0]	2.1[0.5–6.5]	**<0.001**	1.3[0.7–2.8]	**0.038**
**4FU LVEDV, ml**	167[138–193]	164[136–191]	197[170–218]	**<0.001**	187[163–210]	0.082
**4FU LVESV, ml**	80[62–105]	79[61–101]	114[91–140]	**<0.001**	112[89–128]	**0.006**
**4FU LVEF, %**	51[44–57]	51[45–57]	41 [34–50]	**<0.001**	44[34–49]	**0.021**
**4FU IS, % of LVMM**	11.3[4.8–17.9]	10.9[4.2–17.2]	22.2[13.4–28.5]	**<0.001**	20.5[19.4–23.8]	**<0.001**
**4FU LV aneurysm, n (%)**	51(8)	43(8)	8(22)	**0.003**	7 (70)	**<0.001**

CMR = cardiovascular magnetic resonance, IS = infarct size, LVEDV = left ventricular end-diastolic volume, LVESV = left ventricular end-systolic volume, LVMM = left ventricular myocardial mass, MVO = microvascular obstruction; other abbreviations as in Table 1.

At BL CMR, median LVT volume was 0.80 [IQR: 0.31–2.33] cm^3^. In terms of the number of LVTs, 22 patients (59 %) had 1 LVT, whereas 15 patients had fragmented LVTs with 2 fragments (10 patients, 27 %) and 3 fragments (5 patients, 14 %), respectively. All LVTs were located apically, whereas 7 (19 %) were flat wall-adherent, 28 (76 %) intracavitary/mass-like and 2 (5 %) mixed.

At 4FU CMR, median volume of new LVT was 1.47 [IQR: 0.36–4.5] cm^3^. In terms of the number of LVTs, 7 patients (70 %) had 1 LVT, whereas 3 patients (30 %) had fragmented LVTs with 2 fragments (2 patients, 20 %) and 3 fragments (1 patient, 10 %), respectively. All LVTs were located apically, whereas 1 (10 %) was flat wall-adherent and 9 (90 %) were intracavitary/mass-like. Fifty-one patients (8 %) of the overall cohort suffered from LV aneurysm, of which 7 patients (1 %) had late LVT.

The presence of late LVT on CMR was associated with larger BL and 4FU IS (all p < 0.001) and higher rates as well as larger MVO (p = 0.008 and p = 0.038, respectively). Moreover, patients with late LVT had significantly lower LVEF (p = 0.021) and more often LV aneurysm (p < 0.001).

### 3.3 Associates of left ventricular thrombus and risk score development

According to ROC analysis, LAD as culprit vessel (AUC: 0.77, 95 % CI 0.74–0.80; p < 0.001) and 4FU NT-pro-BNP (AUC: 0.79, 95 % CI: 0.75–0.82; p < 0.001) significantly predicted LVT formation (Table 3). Based on the Youden Index, 4FU NT-pro-BNP > 236 ng/l (sensitivity: 100 %; specificity: 55 %), best predicted late LVT formation. Adding NT-pro-BNP above the cut-off to the culprit lesion resulted in a significant continuous NRI (0.1, 0.2) of 0.231 (95 % CI: 0.072–0.389, p = 0.004).

**Table 3:** C-statistics for the prediction of new left ventricular thrombus at 4 months follow-up.

**Variables**	**AUC**	**95 % CI**	**p-value**	**AUC increment**	**ROC comparison**
**Culprit LAD**	0.77	0.74–0.80	**<0.001**	−	−
**4FU NT-pro-BNP**	0.79	0.75–0.82	**<0.001**	0.02	0.712
**4FU NT-pro-BNP > 236 ng/l**	0.78	0.74–0.81	**<0.001**	−0.01	0.790
**Culprit LAD + 4FU NT-pro-BNP > 236 ng/l** **Sensitivity: 100 %** **Specificity: 77 %**	0.89	0.86–0.91	**<0.001**	0.11	**<0.001**
**Criterion: >1**					

AUC = area under the curve, CI = confidence interval, ROC = receiver operating characteristic. Other abbreviations as in Table 1.

Using the score, the population was classified into 2 risk categories: at low risk (0 to 1 point) and at elevated risk (2 points) (Fig. 2). The median risk score of the study population was 1 point [IQR: 0–1]. Of 609 patients, 461 (76 %) were at low risk and 148 (24 %) were at elevated risk. All late LVT occurred in the elevated risk group. The incidence of late LVT was 14.8 % in the elevated risk group (10 of 148 patients), resulting in a number needed to scan of 15 to detect one LVT in this group. In ROC-analysis, the validity of the risk score for the prediction of late LVT formation after STEMI resulted in an AUC of 0.89 (95 % CI 0.86–0.91; p < 0.001, Fig. 2, Table 3). The sensitivity and the specificity of the risk score for the prediction of late LVT after STEMI treated with primary PCI were 100 % and 77 %, respectively.

## 4 Discussion

The present large prospective CMR study evaluated simple and readily available clinical parameters to create a risk score for late LVT prediction after acute STEMI treated with primary PCI. The proposed risk score integrating two variables (LAD as culprit vessel and 4FU NT-pro-BNP) was strongly predictive of late LVT formation as visualized by CMR imaging. All late LVT occurred in the elevated risk group. Contrary, 76 % of the study cohort could be classified as low risk, with no LVT occurring in this group. Consequently, the score is attractive to estimate the risk of late LVT formation after STEMI, which could be helpful to direct the need for advanced imaging with CMR 4 months after the index event in selected patients. Further validation is warranted.

### 4.1 Incidence of left ventricular thrombus and timing of imaging

Different prevalences of early LVT are reported [1]. However, CMR studies investigating the occurrence of late LVT are scarce so far [3,4]. To date, the present work represents the largest CMR study investigating this issue so far.

There are various reasons that potentially explain variations in LVT prevalence including the timing and frequency of imaging, the imaging method used for LVT detection and major changes in STEMI treatment in recent years [6]. In the current investigation, using the reference method for LVT detection, prevalences of 6.1 % and 1.6 % were observed for early and late LVT, respectively. This is in line with a CMR study evaluating early LVT by Pöss et al. including 738 STEMI patients [2]. The incidence rates of LVT occurring at a later time point after STEMI are rare as there are few studies that perform serial post-infarction CMR investigations and clinical FUs are often decentralized. To the best of our knowledge, our cohort is the largest to track LVT formation over time. Observational data from a small CMR study presented an incidence of 6 % of new LVT 4 months after STEMI [4]. A recent study by Bertolin-Boronat et al., which analyzed 590 patients with STEMI, found a 6-month incidence of LVT of 1.2 %, which is similar to our results [27]. As in our study, in most previous CMR trials investigating LVT formation, scanning was performed within a week after STEMI [2,6,[28], [29], [30], [31], [32]]. Nevertheless, early imaging could leave some LVT undetected [33]. To overcome this bias, we performed additional CMR scanning 4 months after STEMI to investigate late LVT formation. In fact, a CMR study by Gellen et al. investigating 265 anterior STEMI patients with an infarcted area of ≥ 10 % of LVMM focused on the optimal time point for LVT detection and revealed a peak incidence at 9 to 12 days after infarction. In this study, 24 patients (9 %) underwent CMR imaging between day 9 to 12 revealing 6 patients with LVT (prevalence = 25 %). Most patients (n = 125, 47 %) underwent CMR between day 3 to 5 after STEMI identifying 12 patients with LVT (prevalence = 9.6 %) [33]. On the other hand, delayed detection of LVT by deferring CMR may result in undertreatment of affected patients. In the present work, the reason for not starting anticoagulation directly in some of the patients with LVT was the delayed receipt of CMR reports. The patients or the attending physician were therefore informed to provide adequate treatment. Further investigations looking at the optimal time point for LVT detection are needed to define the optimal time frame and to investigate the benefit of serial CMR scanning [5].

### 4.2 Predictors of left ventricular thrombi

Several predictors of LVT after STEMI have been described. In previous studies [8,13,22], we investigated predictors of early LVT after STEMI. However, the associates of new LVT after 4 months have not been substantially investigated so far. Almost all LVT are found in patients with anterior location of myocardial infarction [2,29]. LV dysfunction assessed by LVEF is another important factor [2,6,29], which is in concordance with our findings. In addition, a higher risk of LVT in patients with more severe myocardial damage has been described [2]. By using contemporary, multiparametric CMR imaging, we also observed significant associations between infarct severity, MVO and LVT occurrence. Furthermore a great portion of patients with late LVT had LV aneurysm at 4FU CMR, which is in line with previous findings [10]. However, these parameters require comprehensive imaging not always feasible at an outpatient clinic. Myocardial infarction is known to induce a systemic inflammatory activation with release of multiple inflammatory mediators. Inflammation as assessed by circulating CRP concentrations has been described as a good marker of LV dysfunction, more extensive myocardial damage and a predictor of early LVT after STEMI [13,22,34,35]. However, we could not reveal an association between hs-CRP concentrations at 4FU and late LVT formation. On the basis of these findings, we assume that hs-CRP may play a role in early LVT formation. Although our data do not show an association between hs-CRP and late LVT formation, it remains unclear whether there is an association between inflammatory markers other than hs-CRP and late LVT formation. The role of inflammatory processes promoting LVT formation in the chronic stage after STEMI has to be investigated in further studies.

Among others, NT-pro-BNP levels are increased in case of high ventricular filling pressures [36]. Increased NT-pro-BNP concentrations are common in heart failure patients and are useful in risk stratification [37] and guidance for further cardiac investigations [38]. A TTE study investigating 92 patients with LVT revealed that elevated BNP levels are associated with adverse clinical outcome [39]. Furthermore, increased NT-pro-BNP concentrations in the acute phase were found to be predictive for the development of LV aneurysm after STEMI [40]. NT-pro-BNP measured during the acute phase is significantly related to acute and chronic infarct size, worse infarct healing and LV dysfunction after STEMI [[41], [42], [43], [44], [45]]. However, to the best of our knowledge, the association of NT-pro BNP in the chronic phase with the occurrence of late LVT has not been investigated so far. NT-pro-BNP measured in a long-term FU after STEMI might be useful for the estimation of myocardial infarct scar, LV dysfunction and LV aneurysm development as assessed by CMR [46], thus revealing a potential mechanistic link between NT-pro-BNP at 4FU, a marker of myocardial stress, and late LVT formation.

In summary, the pathophysiology of late LVT formation following STEMI involves a complex interplay of mechanical, biochemical and hemostatic factors [1,47]. In patients with large anterior infarctions, particularly those with persistent regional wall motion abnormalities, stasis of blood within dyskinetic or akinetic segments of the left ventricle creates a favorable environment for LVT formation [8,10,48].

Late LVT development might be further influenced by ongoing ventricular remodeling, which can lead to progressive dilation and altered chamber geometry, exacerbating flow disturbances and leading to LV aneurysm formation [1,10], as in our study. Additionally, prolonged endothelial dysfunction and subclinical inflammation, beyond measurable hs-CRP levels, within the infarct zone may sustain a localized prothrombotic state considerably after the acute phase [47].

Some patients may also exhibit persistent hypercoagulability due to systemic inflammatory or neurohormonal activation, further contributing to LVT risk [47]. These pathophysiological mechanisms underscore the importance of continued surveillance in selected high-risk populations, as LVT formation may occur even in the absence of early post-infarction findings. However, it remains speculative whether an early LVT is more thrombogenic than a late onset LVT.

### 4.3 Clinical implication of the risk score

Evaluating patients with STEMI for LVT is challenging. A central problem is the low sensitivity of TTE for LVT detection even when focusing on this specific question. A *meta*-analysis by Bulluck et al. showed that compared with CMR the sensitivity of TTE to verify LVT is only 29 % [1]. The sensitivity of TTE remains low even with the use of contrast agents (64 %) [10]. We revealed a similar sensitivity of non-contrast TTE for the detection of LVT in a previous work [8]. In the present study, 4 of 10 LVT were detected by TTE. To date, focused exams with shortened CMR protocols can be performed in the same time frame as TTE [49], providing superior diagnostic value for LVT detection over TTE. At the same time, routine use of CMR imaging as an initial tool for LVT screening would need immense efforts in the clinical setting. In this regard, the current study has clinical implications. Regarding biomarker measurements at 4 months, only NT-pro-BNP was associated with new LVT in the present work. Furthermore, LAD as culprit lesion location was integrated in the risk score, since all patients with new LVT presented with occluded LAD. The proposed risk score integrates clinical variables beyond cardiovascular imaging parameters that are readily available for almost all of the patients and also accessible to the treating cardiologist in the outpatient clinic during the chronic phase after STEMI. Furthermore, a clear cut-off value was defined for NT-pro-BNP to facilitate the application of the score. Compared with conventional strategies, guiding management based on this risk score could facilitate more appropriate referral to CMR for patients at risk. In fact, a score based referral would have enabled all patients with late LVT to be correctly referred for additional imaging with CMR, while preventing unneeded CMR imaging in the majority of patients (76 %). However, the clinical importance of the echocardiographically-occult LVT identified by risk score-assisted referral to CMR in terms of thromboembolic potential remains unclear. In further consequence, dedicated trials are necessary to validate the effectiveness, generalizability, and value of the proposed risk score-guided diagnostic strategy.

## 5 Limitations

The lack of a validation cohort, the relatively small number of late LVT in our cohort and the observational design represent important limitations of the study. Due to the small event rate, we could not perform a multivariable binary logistic regression analysis to demonstrate the independent predictive value of NT-pro-BNP regarding late LVT detection. Further research and validation are therefore needed. On the other hand, this study represents a very comprehensive evaluation of clinical and laboratory imaging information in a large cohort of STEMI patients. Moreover, the score was developed in stable patients (Killip class < 3) with acute and first time STEMI. Notably, the vast majority of STEMI patients presents with Killip class < 3 [50]. The score is thus not applicable to unstable STEMI patients or in case of previous myocardial infarction and needs further validation in this setting as well. However, the inclusion of patients with prior myocardial infarctions or previous PCI could introduce bias, as these patients may already have thrombi from earlier infarcts, which could confound the assessment of LVT formation related to the current event. T2* mapping for detection of intramyocardial hemorrhage was not available for a significant portion of patients and we can therefore not assess the potential association between intramyocardial iron deposition and LVT post-STEMI. At 4-month follow-up, only those patients with LVT diagnosed by CMR were referred to a TTE (blinded examiner) within the hospital for an internal diagnostic accuracy assessment of TTE for thrombus detection.

## 6 Conclusions

In patients treated with PCI for acute STEMI, a new risk score based on simple clinical factors (LAD as the culprit vessel and NT-proBNP > 236 ng/l after 4 months) may help identify those at elevated risk of late LVT and guide the use of CMR imaging. Its practical utility remains to be confirmed in larger, prospective cohorts.

## CRediT authorship contribution statement

**Magdalena Holzknecht:** Writing – original draft, Visualization, Project administration, Methodology, Investigation, Funding acquisition, Formal analysis, Data curation, Conceptualization. **Christina Tiller:** Writing – review & editing, Formal analysis, Data curation. **Ivan Lechner:** Writing – review & editing, Formal analysis, Data curation. **Fritz Oberhollenzer:** Writing – review & editing, Formal analysis, Data curation. **Alex Kaser:** Writing – review & editing, Formal analysis, Data curation. **Martin Reindl:** Writing – review & editing, Formal analysis, Data curation. **Felix Troger:** Writing – review & editing, Data curation. **Mathias Pamminger:** Writing – review & editing, Data curation. **Axel Bauer:** Writing – review & editing, Supervision, Resources, Formal analysis. **Bernhard Metzler:** Writing – review & editing, Supervision, Resources, Project administration, Funding acquisition, Formal analysis. **Sebastian Johannes Reinstadler:** Writing – review & editing, Validation, Project administration, Investigation, Formal analysis, Conceptualization. **Agnes Mayr:** Writing – original draft, Validation, Supervision, Project administration, Methodology, Investigation, Formal analysis, Conceptualization.

## Declaration of competing interest

The authors declare that they have no known competing financial interests or personal relationships that could have appeared to influence the work reported in this paper.
